# A Japanese patient with hereditary spastic paraplegia with a rare *KIF5A* nonsense variant

**DOI:** 10.1038/s41439-025-00313-3

**Published:** 2025-06-02

**Authors:** Shiroh Miura, Seria Suenaga, Hana Goto, Zhaonan Wang, Akane Makino, Luoming Fan, Kensuke Senzaki, Masayuki Ochi, Yasumasa Ohyagi, Hiroki Shibata

**Affiliations:** 1 Department of Neurology and Geriatric Medicine, Ehime University Graduate School of Medicine, Toon, Japan; 2 Division of Genomics, Medical Institute of Bioregulation, Kyushu University, Fukuoka, Japan

**Keywords:** Neurodegeneration, Motor neuron disease

## Abstract

Spastic paraplegia (SPG)10 is an autosomal dominant SPG caused by kinesin family member 5A (*KIF5A*) gene variants. We describe a Japanese patient with SPG whose deceased mother and maternal uncle also exhibited SPG. Exome analysis identified a rare *KIF5A* nonsense variant (NM_004984.4:c.2590C>T (p.Arg864Ter)) in the patient, regarded as pathogenic. As *KIF5A* mRNA expression was significantly decreased compared with that of a healthy control, the variant was deemed causative of SPG.

Hereditary spastic paraplegia (SPG) comprises a group of clinically and genetically heterogeneous disorders, characterized mainly by progressive lower limb spasticity and weakness as a result of distal degeneration of corticospinal tract axons^1^. SPG10 (MIM; Mendelian Inheritance in Man: 604187) is an autosomal dominant hereditary SPG caused by variants in the kinesin family member 5A gene (*KIF5A*, MIM: 602821), also known as the kinesin heavy chain gene (*KHC*), which is located at chromosome 12q13 (refs. ^2–4^). *KIF5A* RNA is expressed exclusively in brain tissue^5^. KIF5A is associated with RNA transport in dendrites and consists of an N-terminal motor domain, a coiled-coil hinge domain and a C-terminal cargo-binding domain^6^.

A 60-year-old male Japanese patient initially noticed muscle cramping in his calves while attending junior high school and exhibited pollakiuria while in high school. He subsequently experienced gait disturbance at the age of 20 and underwent catheter ablation for paroxysmal atrial fibrillation at 60 years of age. His mother and maternal uncle had spastic gaits. A neurological examination showed hyperreflexia, spasticity, weakness in his lower extremities and bilateral Babinski reflexes. He demonstrated mild impaired vibration sense with dominancy in the distal portion of the lower limbs. His superficial sensation and position sense were intact. Cognitive impairment was not observed. A cranial nerve examination found no abnormalities, and there was no sign of cerebellar ataxia. He did not have pes cavus. Serum anti-human T-lymphotropic virus-1 antibody levels were negative, and the standard cerebrospinal fluid examination was normal. Nerve conduction studies on the right median, ulnar, tibial and sural nerves revealed normal motor conduction velocities, distal compound muscle action potential amplitudes and sensory conduction velocities. The distal motor latency was slightly prolonged in the median nerve, and sensory nerve action potential amplitudes were mildly reduced in all nerves tested. Upon examination of motor evoked potentials, the bilateral central motor conduction time was normal, and no abnormalities were observed in brain or cervical magnetic resonance imaging. In summary, the patient showed slowly progressive SPG with bladder dysfunction and peripheral neuropathy, which is compatible with SPG10^7^.

We first confirmed absence of an exonic copy number variant in *SPAST* (MIM: 604277) by fragment analysis using the SALSA MLPA Reagent Kit (MRC Holland) on the patient’s DNA. We detected 20,640 single-nucleotide variants by exome sequencing of the proband. We prioritized 17 genes known to be responsible for autosomal dominant SPG: *ALDH18A1* (MIM: 138250), *ATL1* (MIM: 606439), *BSCL2* (MIM: 606158), *CPT1C* (MIM: 608846), *HSPD1* (MIM: 118190), *KIF1A* (MIM: 601255), *KIF5A* (MIM: 602821), *KPNA3* (MIM: 601892), *NIPA1* (MIM: 608145), *REEP1* (MIM: 609139), *REEP2* (MIM: 609347), *RTN2* (MIM: 603183), *SLC33A1* (MIM: 603690), *SPAST* (MIM: 604277), *UBAP1* (MIM: 609787), *WASHC5* (MIM: 610657) and *ZFYVE27* (MIM: 610243). Functional nonsynonymous, nonsense, frameshift, in-frame insertion/deletion and splice site variants with minor allele frequencies <0.0001 were selected using gnomAD (https://gnomad.broadinstitute.org), and a *KIF5A* nonsense variant, NM_004984.4:c.2590C>T (p.Arg864Ter) (rs1882578572), was identified in the patient. This was validated by Sanger sequencing using forward primer 5′-AGCTGGTACGTGACAATGCA-3′ and reverse primer 5′-CTCCTTGGCCTCCTTCAGTG-3′. The patient was confirmed to be heterozygous for the variant, consistent with the autosomal dominant mode of the predicted disease inheritance (Fig. 1).

**Fig. 1:**
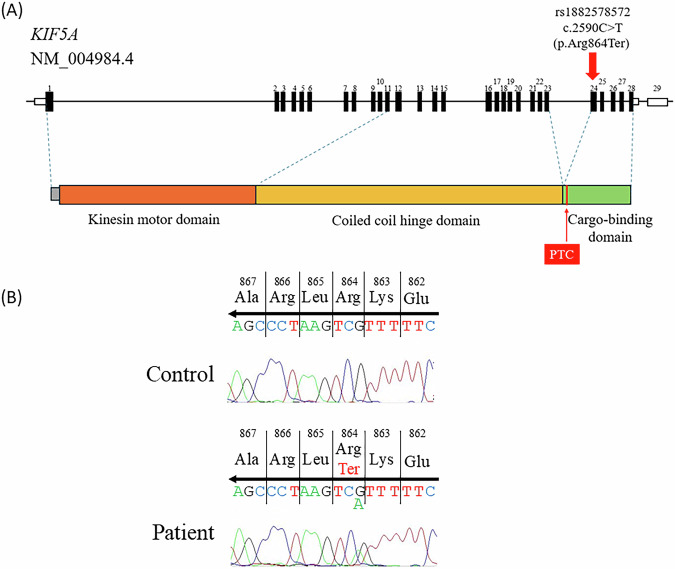
The nonsense variant in *KIF5A* in the Japanese SPG patient. **A** The genomic structure of *KIF5A*. Filled and open boxes represent coding and noncoding regions of transcript NM_004984.4, respectively. The nonsense variant NM_004984.4:c.2590C>T (p.Arg864Ter) is shown by a red arrow. *KIF5A* consists of 29 exons; exons 1–11, 11–23 and 24–28 encode the kinesin motor domain, coiled-coil hinge domain and cargo-binding domain, respectively. PTC, premature termination codon. **B** An electropherogram of the region of the variant NM_004984.4:c.2590C>T (p.Arg864Ter) in the patient and a healthy control. A heterozygous G-to-A substitution was detected at position 2590 in the patient, causing a premature termination at codon 864. The direction of translation is shown by the arrows.

This nonsense variant was previously reported in a Greek patient with sporadic SPG accompanied by sensorimotor axonal neuropathy, lower limb hemiatrophy and pes cavus^8^. It is located in *KIF5A* exon 24 and results in truncation of the KIF5A protein at the C-terminal cargo-binding domain. A significant decrease in *KIF5A* expression was detected in the patient’s peripheral blood, compared with that of a healthy control, probably resulting from nonsense-mediated decay (Fig. 2). According to ACMG–AMP (American College of Medical Genetics and Association of Molecular Pathology) guidelines^9,10^, the variant is classified as ‘pathogenic’, meeting PVS1, PS4, PM1, PM2 and PM4 criteria.

**Fig. 2:**
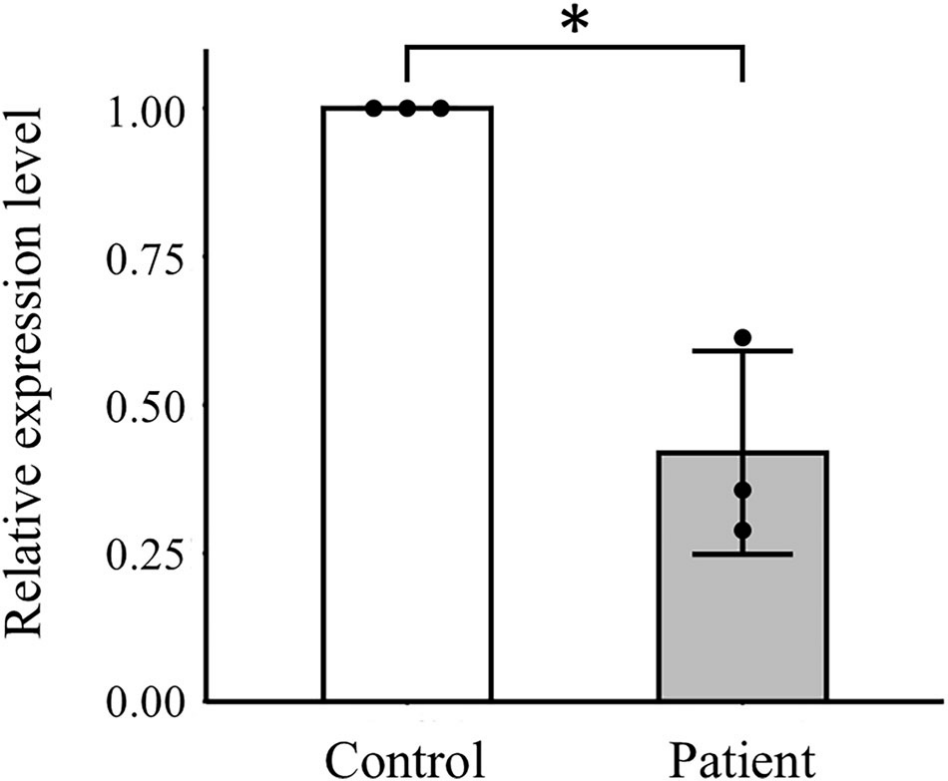
Quantitative determination of *KIF5A* mRNA expression levels. *KIF5A* mRNA was quantified by real-time quantitative PCR using the forward primer: 5′-CGGTGGCGCAATGGAGA-3′ and reverse primer: 5′-CCTCGTATTTCTGCCGCTCC-3′. The experiment was performed in triplicate. Values represent the mean ± s.d. normalized to *GAPDH* mRNA levels. Relative expression levels were calculated by the ΔΔCT method because of the quantitative limitation of the patient sample. *P = 0.0135.

The variant is expected to be translated into a truncated protein, lacking the C-terminal cargo-binding domain that is known to be essential for sliding and bundling of microtubule, and autoinhibition as well as cargo binding^11–13^. Although functional variants located in this domain are frequently associated with amyotrophic lateral sclerosis and neonatal intractable myoclonus syndrome (MIM: 617235)^14^, both our patient and the previous patient with the nonsense variant exhibited SPG with neuropathy, which is compatible with SPG10. The dominant negative effect of the truncated protein product and/or haploinsufficiency of the functional protein caused by nonsense-mediated decay are likely to be involved in the pathogenesis of SPG in the pedigree.

In this study, we conclude that the *KIF5A* nonsense variant NM_004984.4: c.2590C>T (p.Arg864Ter) is causative of SPG10.

## HGV datbase

The relevant data from this Data Report are hosted at the Human Genome Variation Database at: 10.6084/m9.figshare.hgv.3500.
